# Effect of the monoclonal antibody TRC105 in combination with Sunitinib on renal tumor derived endothelial cells

**DOI:** 10.18632/oncotarget.25206

**Published:** 2018-04-27

**Authors:** Alessia Brossa, Lola Buono, Benedetta Bussolati

**Affiliations:** ^1^ Department of Molecular Biotechnology and Health Sciences, University of Torino, Turin, Italy

**Keywords:** TRC105, Sunitinib, tumor stem cells, angiogenic therapy, renal cell carcinoma

## Abstract

Anti-angiogenic therapy is an important strategy to limit growth, development and expansion of solid tumors. However, resistance to VEGF-targeting agents may develop, due to activation of alternative pro-angiogenic pathways, indicating the need of multiple target strategy. Here we obtained tumor endothelial cells (TEC) either from total renal carcinomas or from renal cancer stem cells (CSC-TEC) and we tested the effect of a CD105 targeting monoclonal antibody, TRC105, alone or in association with anti-VEGF drugs. We demonstrated that TRC105 impaired the ability of TEC and CSC-TEC to organize in tubular structures, whereas it did not limit proliferation or survival. The combination of TRC105 with different anti-angiogenic drugs showed a synergistic effect of TRC105 only in combination with the tyrosine kinase inhibitor Sunitinib. In particular, TRC105 plus Sunitinib reduced tubulogenesis, proliferation and survival of CSC-TEC and tumor-derived TEC in a similar manner. At a molecular level, we showed that the combination of TRC105 and Sunitinib induced the phosphorylation of Smad 2/3 to promote endothelial cell death. Moreover, TRC105 enhanced the inhibitory effect of Sunitinib on VEGF signaling and reduced VEGFR2-Akt-Creb activation, suggesting a molecular cooperation between the two drugs. Our results highlight that the combined inhibition of VEGF and TGF-β pathway may have a potential use in renal cell carcinoma therapy.

## INTRODUCTION

Angiogenesis is fundamental for solid tumor growth, development, and expansion. Tumor vessels differ from normal ones in the structural organization of the endothelium, suggesting that tumor vasculature originates by mechanisms other than the recruitment of blood vessels from pre-existing ones in adjacent tissues. One possible proposed mechanism is intra-tumor vasculogenesis [1–3], due to endothelial differentiation of cancer stem cells (CSC), a subpopulation of tumor cells with stem properties [4–7]. *In vitro* CSC are quiescent cells, capable of self-renewal and differentiation into different cell types, while *in vivo* they show tumor initiating properties, as CSC-derived tumors contain differentiated tumor cells, CSC, and endothelial cells [4, 8, 9]. Several groups have demonstrated that CSC differentiate into endothelial cells and pericytes and contribute to tumor vasculogenesis [9–17]. Since CSC are responsible for the maintenance and growth of tumors, they may represent a target for cancer therapy [18]. Both in healthy kidney and renal carcinomas the presence of mesenchymal stem cells expressing CD105 was observed [8]. We previously isolated from human renal carcinomas CD105^+^ CSC, that were able to acquire an endothelial phenotype both *in vitro* and *in vivo* [8, 19]. CSC-derived tumor endothelial cells (TEC) display increased pro-angiogenic features, including overexpression of pro-angiogenic receptors and survival [3]. This may lead to increased resistance to anti-angiogenic therapies. In addition, the inhibition of VEGF pathway may lead to VEGF-R2 and PDGFR-β overexpression after treatment discontinuation and to tumor VEGF-independency [20]. It was therefore proposed that prolonged anti-angiogenic therapies may contribute to therapy resistance and maintain the hypoxia-dependent CSC stemness, as observed in breast cancer xenotrapiants [21].

CD105, a TGF-β co-receptor over-expressed on proliferating endothelial cells, plays a fundamental regulatory role in endothelial cell activation. It is overexpressed on endothelial cells of healing wounds, developing embryos, inflammatory tissues, and solid tumors, being a marker of activated endothelium, since its vascular expression is limited to proliferating cells [22]. Dense CD105 expression on vessels is correlated with poor prognosis in many solid tumors including breast, lung, prostate, kidney, liver, and colon [23–25]. TRC105 (Carotuximab) is a chimeric immunoglobulin G1 monoclonal antibody that binds CD105. TRC105 is currently being studied in a Phase 3 trial in combination with Pazopanib in advanced angiosarcoma (NCT02979899), and has been tested in multiple Phase 1 and 2 clinical trials for the treatment of solid tumors in combination with different VEGF inhibitors [26–29]. However, the effect of TRC105 on different mechanisms of tumor vascularization, such as intra-tumor vasculogenesis, remains unknown. In the present study, we tested TRC105 alone or in combination with different anti-angiogenic drugs approved for renal cell carcinoma therapy, on both TEC lines and CSC derived TEC isolated from renal cell carcinoma specimens. Finally, we investigated the molecular mechanisms involved in the synergistic effect of TRC105 and tyrosine kinase inhibition.

## RESULTS

### Endothelial differentiation of CSC

**Figure 1:**
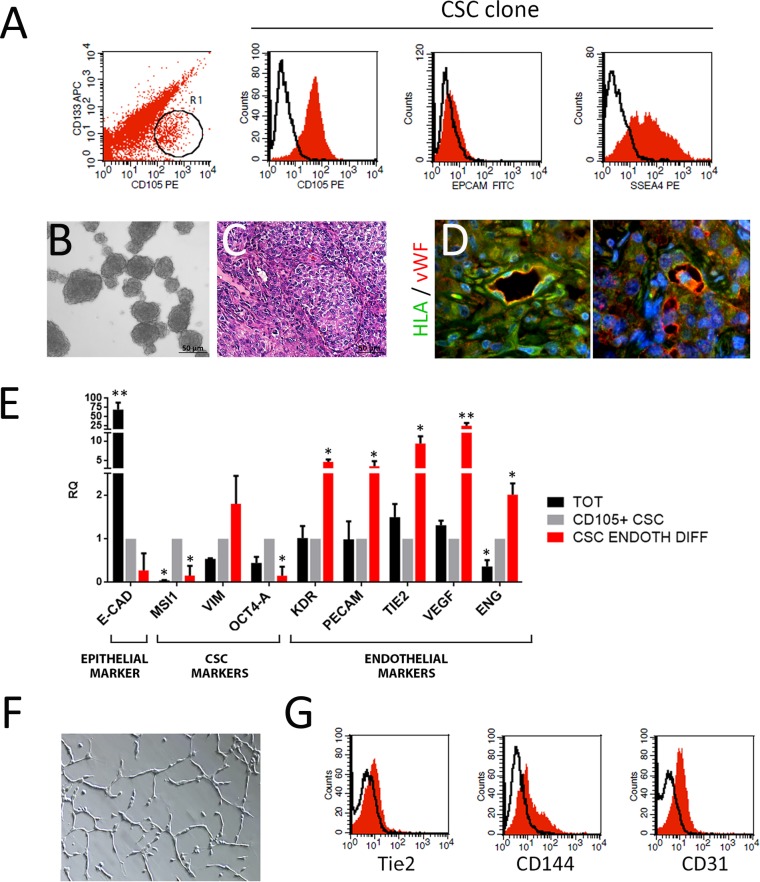
Endothelial differentiation of CSC **(A)** Flow cytometry analysis of total tumor cell population after isolation and of CD105^+^/CD133^-^ CSC. R1 represents CD105+/CD133- subpopulation from which the CSC clone was isolated. The red area shows binding of the specific antibody and the dark line the isotypic control. **(B)** Representative micrograph of CSC-derived spheres. Original magnification x200. **(C)** Hematoxylin and eosin staining of CSC-derived tumors. Original magnification x200. **(D)** Immunofluorescence micrographs of CSC-derived tumors showing the coexpression of HLA (green) and vWF (red) in vessels infiltrating the tumor. Original magnification x400. **(E)** Real time analysis showing the expression of E-Cadherin (ECAD), Musashi (MSI), Vimentin (VIM), OCT4-A, KDR, PECAM, TIE2, VEGF and ENG of the total tumor population (TOT) and endothelial-differentiated CSC (CSC ENDOTH DIFF) respect to undifferentiated CD105^+^/CD133^-^ CSC clone (CD105+ CSC). Data are expressed as RQ and normalized vs GAPDH and to CD105+ CSC. ^*^=p<0.05 and ^**^=p<0.001 vs CD105+ CSC. **(F)** Representative micrograph of tubular structures formed by endothelial-differentiated CSC. Original magnification x100. **(G)** Flow cytometry analysis of endothelial differentiated CSC showing the expression of TIE2, Ve-Cadherin (CD144) and PECAM (CD31). The red area shows binding of the specific antibody and the dark line the isotypic control.

Renal cell carcinoma stem cells (CSC) were isolated from a nephrectomy specimen of renal clear cell canrcinoma, as previously described [8, 30, 31]. CD105^+^ CSC were sorted, cloned and characterized as tumor stem cells based on the following criteria: *1*) were clonogenic, *2*) expressed stem cell markers and lacked differentiation markers, *3*) could differentiate *in vitro* into endothelial cell types, and *4*) could generate *in vivo* serially transplantable tumors. These tumors, despite being derived from clones expressing mesenchymal markers, were epithelial carcinomas. CD105^+^/CD133^-^ cells, representing the CSC population, were less than 10% of the total tumor population (Figure 1A). One CD105^+^/CD133^-^ clone was selected, and the presence of CD105^+^ was confimed by FACS analysis, as was the presence of the stem cell marker SSEA4 and the absence of the epithelial marker EPCAM (Figure 1A). Compared to the total tumor population, CD105^+^ CSC clone expressed the stem cell related genes Musashi (MSI), Vimentin (VIM) and OCT4-A, and lacked the expression of the epithelial marker E-CAD (Figure 1E). Moreover, these cells were able to grow as spheres in non-adherent culture conditions (Figure 1B). To test their tumorigenic ability, 100 cells were injected subcutaneously in SCID mice. After 2 weeks, tumors were palpable in 100% of mice. In mice derived tumors (Figure 1C), we observed the presence of human vessels infiltrating the tumor (Figure 1D) as HLA/VWF expressing cells, suggesting an endothelial differentiation of CD105^+^ CSC. Endothelial differentiation of CD105^+^ CSC was observed *in vitro* by culturing the cells in hypoxia for 14 days, as previously described [31].

CSC-derived endothelial cells acquired the expression of the endothelial markers Ve-Cadherin (CD144), KDR (VEGF-R2), PECAM (CD31), TIE2 and VEGF, and lost the expression of the stem cell marker MSI and OCT4-A (Figure 1E and 1G).

In addition, when differentiated towards an endothelial state, CSC acquired the ability to form tubular-like structures when plated in Matrigel (Figure 1F).

### TRC105 impairs *in vitro* angiogenesis but not proliferation and survival of renal tumor endothelial cells

**Figure 2:**
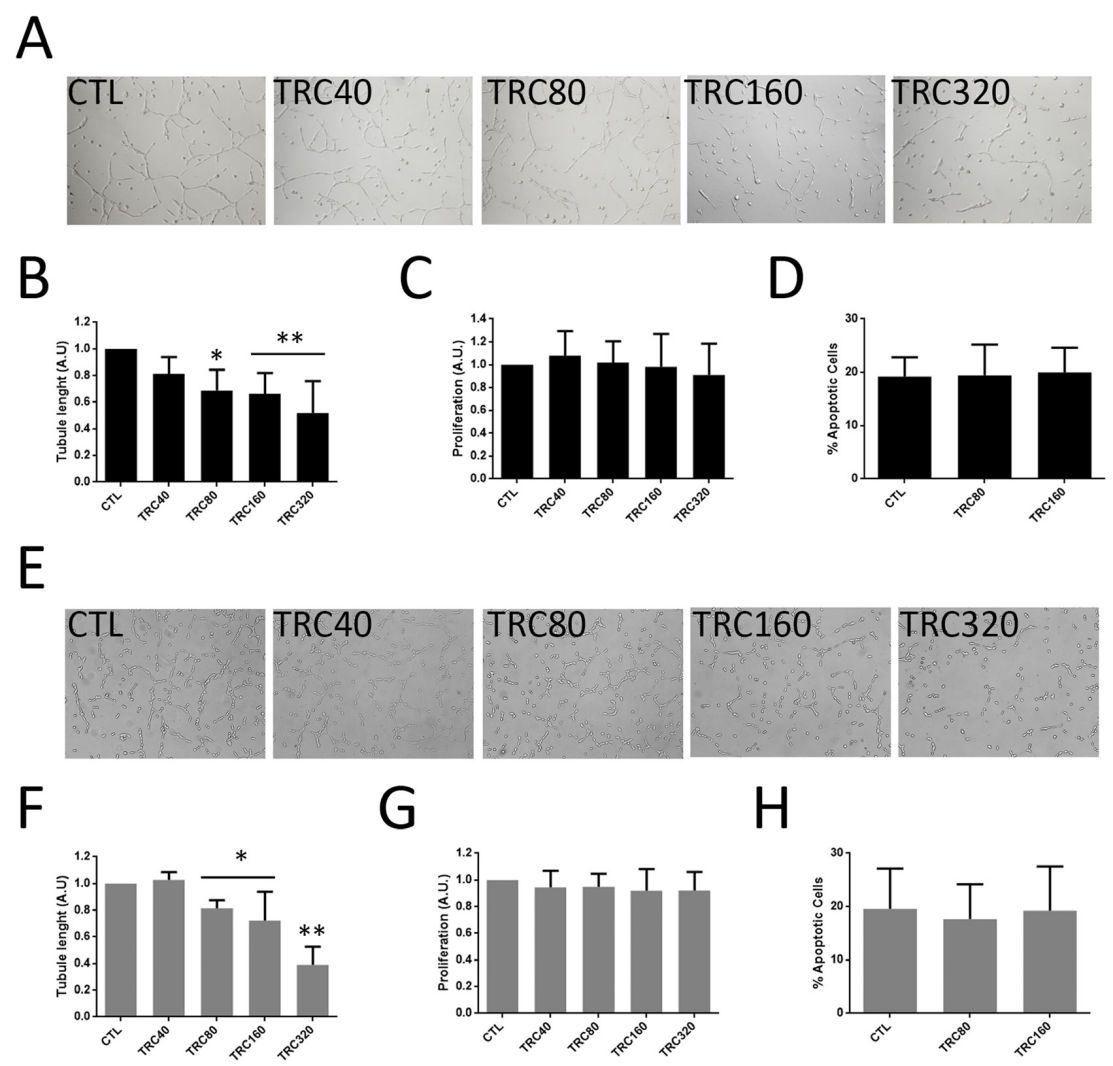
Tube formation, proliferation and survival of CSC-TEC and TEC treated with TRC105 **(A and E)** Representative micrograph of tubular structures formed by CSC-TEC (A) or TEC (E) at increasing levels of TRC105 (TRC40-TRC320, corresponding to TRC105 from 40 to 320 μg/ml). Original magnification x100. **(B and F)** Tube length of CSC-TEC (B) and TEC (F) at different doses (40-320 μg/ml) of TRC105. Data are represented as mean ± SD of at least three independent experiments normalized to untreated cells (CTL). ^*^=p<0.05 and ^**^=p<0.001 vs CTL. **(C and G)** Proliferation levels of CSC-TEC (C) and TEC (G) at different doses (40-320 μg/ml) of TRC105. Data are represented as mean ± SD of at least three independent experiments normalized to CTL. **(D and H)** Percentage of apoptotic CSC-TEC (D) and TEC (H) treated with two different doses of TRC105 (80 and 160 μg/ml). Data are represented as mean ± SD of at least three independent experiments.

We evaluated the effect of different doses of the monoclonal antibody TRC105 on proliferation, survival and the ability to form tubular-like structures on tumor endothelial cells (TEC, previously isolated and characterized [32]) and cancer stem cells differentiated in endothelial cells (CSC-TEC) (Figure 2). TRC105 significantly inhibited tubule-like structure formation both of CSC-TEC (Figure 2A and 2B) and of TEC (Figure 2E and 2F) in a dose-dependent manner. However, TRC105 did not inhibit proliferation (Figure 2C and 2G) or survival (Figure 2D and 2H) of CSC-TEC or TEC. TRC105 had no effect on CD105^+^ undifferentiated CSC nor on CD105^-^ renal carcinoma cell lines (Supplementary Figure 1).

### The combination of TRC105 with Sunitinib is effective in inhibiting proliferation, survival and *in vitro* angiogenesis

**Figure 3:**
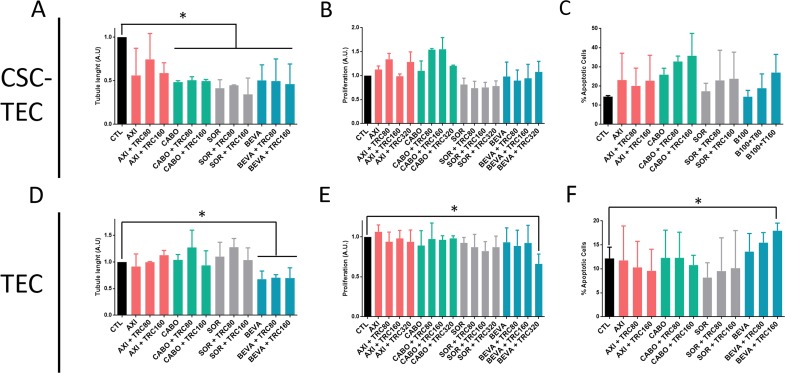
Tube formation, proliferation and survival of CSC-TEC and TEC treated with TRC105 in combination with different anti-angiogenic drugs **(A and D)** Tube length of CSC-TEC (A) and TEC (D) at increasing doses of TRC (TRC80-TRC320, corresponding to TRC105 from 80 to 320 μg/ml) in combination with Axitinib (AXI), Cabozantinib (CABO), Sorafenib (SOR) (all 1 μM) and Bevacizumab (BEVA) (100 μg/ml). **(B and E)** Proliferation levels of CSC-TEC (B) and TEC (E) at escalating doses of TRC105 (80-320 μg/ml) in combination with AXI, CABO, SOR and BEVA. Data are represented as mean ± SD of at least three independent experiments normalized to CTL and to 1. **(C and F)** Percentage of apoptotic CSC-TEC (C) and TEC (F) treated with AXI, CABO, SOR and BEVA alone or in combination with different doses of TRC105 (80-160 μg/ml). Data are represented as mean ± SD of at least three independent experiments normalized to untreated cells (CTL). ^*^=p<0.05 vs CTL.

**Figure 4:**
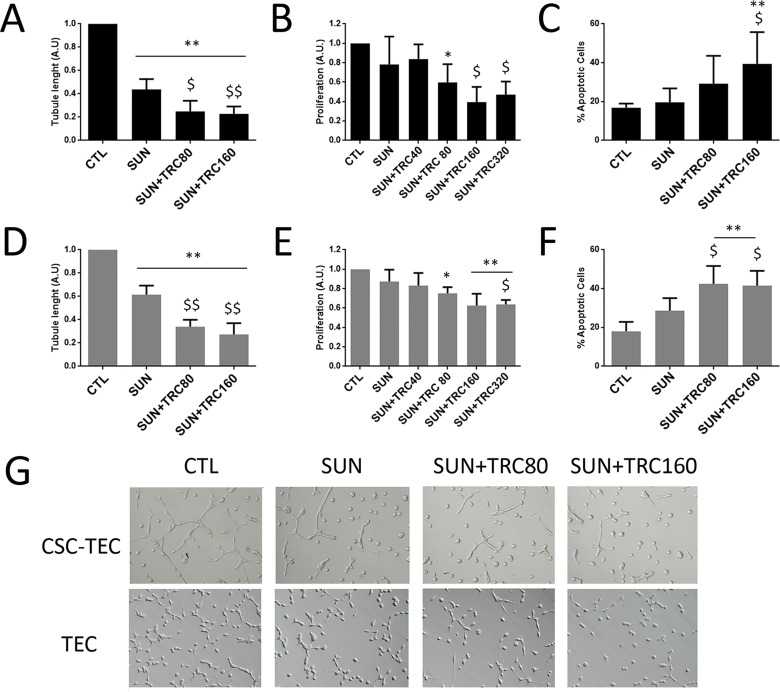
TRC105 in combination with Sunitinib inhibits tube formation, proliferation and survival of CSC-TEC and TEC **(A and D)** Tube length of CSC-TEC (A) and TEC (D) at increasing levels of TRC105 (TRC80 and TRC160, corresponding to TRC105 80 and 160 μg/ml) in combination with tyrosine kinase Sunitinib (SUN, 0.1μM). Data are represented as mean ± SD of at least three independent experiments normalized to untreated cells (CTL). ^**^=p<0.001 vs CTL; ^$^=p<0.05 and ^$$^=p<0.001 vs SUN. **(B and E)** Proliferation levels of CSC-TEC (B) and TEC (E) at escalating doses of TRC105 (40-320 μg/ml) in combination with SUN (1μM). Data are represented as mean ± SD of at least three independent experiments normalized to CTL and to 1. ^*^=p<0.05 and ^**^=p<0.001 vs CTL; ^$^=p<0.05 vs SUN. **(C and F)** Percentage of apoptotic CSC-TEC (C) and TEC (F) treated with SUN alone (1μM) or in combination with two different doses of TRC105 (80-160 μg/ml). Data are represented as mean ± SD of at least three independent experiments. ^**^=p<0.001 vs CTL; ^$^=p<0.05 vs SUN. **(G)** Representative micrograph of tubular structures formed by CSC-TEC or TEC treated with SUN (0.1μM) in combination with increasing levels of TRC (80-160 μg/ml). Original magnification x100.

In order to test if the combination of TRC105 would increase the effect of other anti-angiogenic drugs, we tested four anti-angiogenic drugs in combination with TRC105. A dose response curve of the selected anti-angiogenic drugs was performed and a dose with non significant apoptotic effect was chosen (not shown). We evaluated Axitinib, Cabozantinib, Sorafenib, Bevacizumab (Figure 3) and Sunitinib (Figure 4) alone and in combination with different doses of TRC105 on tubular-like structure formation (Figure 3A and 3D), proliferation (Figure 3B and 3E) and survival (Figure 3C and 3F). Synergy was not observed by addition of TRC105 to Axitinib, Cabozantinib and Sorafenib, as evaluated on tube formation, proliferation or apoptosis. Bevacizumab showed a partial synergistic effect with TRC105, at the maximum TRC105 concentration only (320 μg/ml), as the combination decreased proliferation (Figure 3E) and induced apoptosis (Figure 3F) on TEC. TRC105 did not increase the inhibitory effect of Bevacizumab on tube formation of both CSC-TEC and TEC. Instead, Sunitinib alone (0.1 μM, Figure 4) reduced tube formation, and the effect was significantly increased in combination with TRC105 (Figure 4A, 4D and 4G). Proliferating activity of CSC-TEC and TEC decreased in a dose-dependent manner when TRC105 was combined with Sunitinib (1 μM) (Figure 4B and 4E). In addition, the percentage of apoptotic cells significantly increased only when both CSC-TEC and TEC were treated with Sunitinib (1 μM) in combination with TRC105 (Figure 4C and 4F). Moreover, the same additive effect was observed on normal human humbilical cord-derived endothelial cells (HUVEC), that showed a significant reduction of tube lenghts, proliferation and survival when incubated with Sunitinib and TRC105 (Supplementary Figure 2). No synergistic effect on proliferation and apoptosis was observed at the lower Sunitinib dose (0.1 μM) on all tumor and normal endothelial cells (not shown). Furthermore, TRC105 did not exhert an additive effect on Sunitinib-induced apoptosis of CSC or primary renal carcinoma cell lines (Supplementary Figure 1).

### TRC105 and Sunitinib combination promotes cell apoptosis by increasing Smad 2/3 phosphorylation

**Figure 5:**
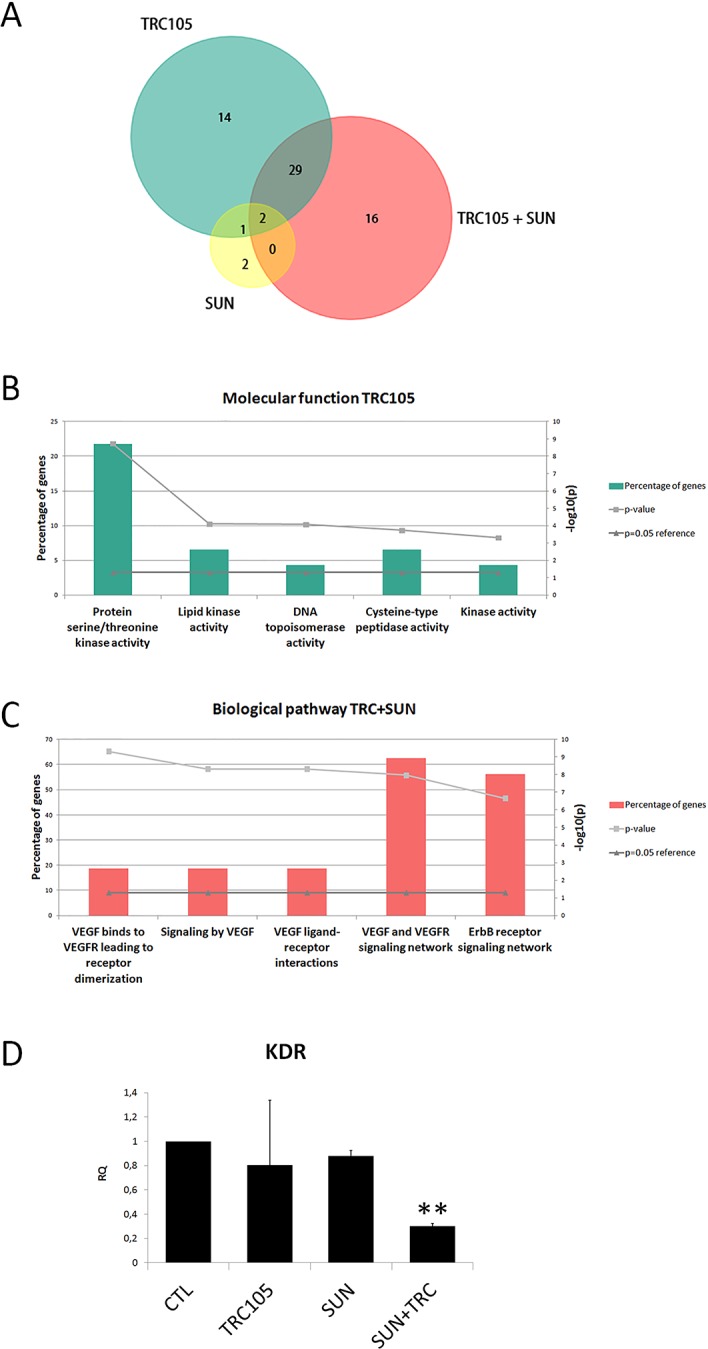
Molecular analysis of CSC-TEC treated with TRC105 and Sunitinib alone or in combination **(A)** Funrich analysis of regulated genes of CSC-TEC treated with TRC105 (160 μg/ml), Sunitinib 0.1μM (SUN) or the combination of the two drugs (TRC105+SUN). **(B)** Molecular function analysis of the genes regulated by the single therapy treatment with TRC105 on CSC-TEC. **(C)** Biological pathway analysis of CSC-TEC genes regulated by the combination of TRC105 and SUN. **(D)** Real time analysis of KDR mRNA expression in CSC-TEC treated with TRC105 alone, SUN, or the combination of both. ^**^=p<0.001 vs CTL.

We analyzed the effect of TRC105 alone or in combination with an ineffective dose of Sunitinib on the modulation of the expression of 86 drug targets genes on CSC-TEC. TRC105 alone induced the regulation of 46 genes, while CSC-TEC treatment with Sunitinib as a single therapy induced the regulation of 5 genes (Figure 5A and Supplementary Table 1). Analyzing the molecular function of single drug treatment with TRC105, we observed it affected the regulation of several serine/threonine kinases (Figure 5B). In addition, when cells were treated with the combination of the two drugs, additional 16 genes were regulated, including growth factor receptors and protein kinases (Figure 5A and Supplementary Table 1). Biologic pathway analysis of genes regulated by TRC105 in combination with Sunitinib (47 genes) showed the modulation of genes mainly involved in the VEGF signaling pathway (Figure 5C). We confirmed the VEGF pathway modulation by analysis of the KDR receptor following combined treatment with Sunitinib and TRC105. We observed a significant down-regulation of KDR in cells treated with the combination of the two anti-angiogenic drugs (Figure 5D).

**Figure 6:**
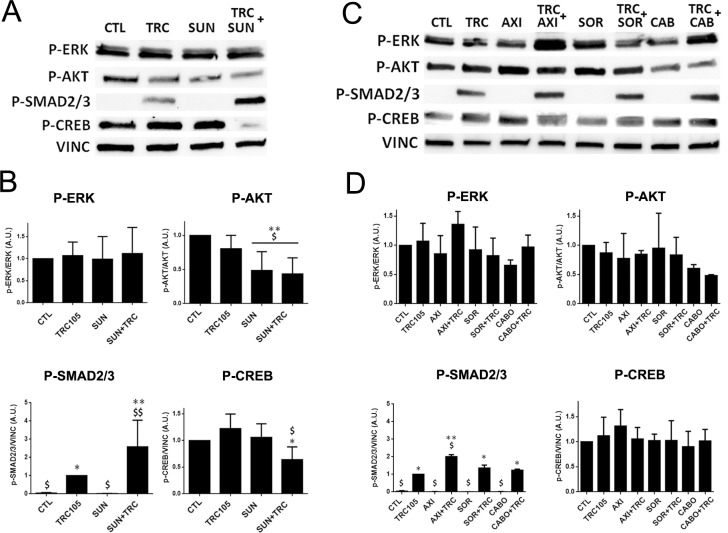
Western blot analysis on CSC-TEC treated with TRC105 alone or in combination with Axitinib, Sorafenib, Cabozantinib and Sunitinib **(A)** Representative micrographs of western blot on CSC-TEC treated with TRC105 (160 μg/ml) alone or in combination with Sunitinib (1 μM), using antibodies against p-Erk, p-AKT, p-Smad 2/3 and p-Creb. **(B)** Western blot analysis showing the quantification of the protein levels of CSC-TEC treated with TRC105 (160 μg/ml) alone or in combination with Sunitinib (1 μM), normalized to Vinculin and to CTL. The relative expression of p-Erk and p-Akt were also normalized to basal Erk and Akt. ^*^=p<0.05 and ^**^=p<0.001 vs CTL; ^$^=p<0.005 and ^$$^=p<0.001 vs TRC. **(C)** Representative micrographs of western blot on CSC-TEC treated with TRC105 (160 μg/ml) alone or in combination with Axitinib (AXI), Sorafenib (SOR) and Cabozantinib (CABO) (all 1 μM), using antibodies against p-Erk, p-AKT, p-Smad 2/3 and p-Creb. **(D)** Western blot analysis showing the quantification of the protein levels of CSC-TEC treated with with TRC105 (160 μg/ml) alone or in combination with Axitinib (AXI), Sorafenib (SOR) (all 1 μM), normalized to Vinculin and to CTL. The relative expression of p-Erk and p-Akt were also normalized to basal Erk and Akt. ^*^=p<0.05 and ^**^=p<0.001 vs CTL; ^$^=p<0.005 vs TRC.

In order to better understand the molecular pathways involved in the synergistic effect of TRC105 and Sunitinib, we evaluated the activation of VEGF and TGF-β intracellular pathways. In particular, we studied the levels of p-Smad 2/3, activated by TGF-β signalling, and of p-Erk, p-Akt and p-Creb, activated by VEGF (Figure 6). Smad 2/3 phosphorylation was highly induced by TRC105 alone and further increased by the combination with the tyrosine kinase inhibitor Sunitinib (Figure 6A and 6B), while levels of p-Smad 1/5 did not vary in the presence of the TRC105 and Sunitinib (not shown). Furthermore, p-Akt levels were lower in the presence of TRC105 and significantly decreased when CSC-TEC were treated with Sunitinib and the combination of both (Figure 6A and 6B). As a consequence, the phosphorylation of the transcription factor Creb was decreased by the combination of the two drugs (Figure 6A and 6B).

**Figure 7:**
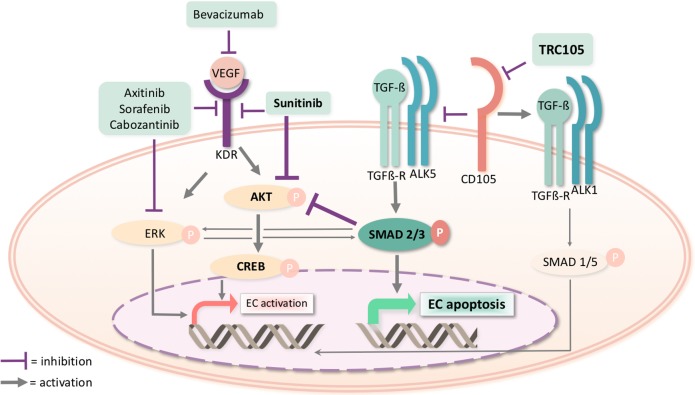
Molecular pathways involved in the pharmacological inhibition of CD105 by TRC105 and of KDR by Sunitinib In endothelial cells (EC), TRC105 blocks CD105 favouring the TGF-ß receptor/ALK5 complex signaling. This complex phosphorylates and activates Smad 2/3 which can translocate in the nucleus leading to endothelial cell apoptosis. Sunitinib inhibits the signaling pathway of VEGF receptor 2 (KDR), resulting in a reduced Akt activity. In the presence of both drugs, Akt is synergistically inhibited by Sunitinib and by Smad 2/3 resulting in a reduced phosphorylation of the transcription factor Creb, involved in cell activation. Other tyrosine kinase inhibitors (Axitinib, Sorafenib and Cabozantinib), show a predominant modulation of the Erk pathway and do not affect Creb phosphorylation. No synergistic effect of TRC105 is present in combination with Axitinib, Sorafenib and Cabozantinib on VEGF receptor signaling pathways.

We also evaluated VEGF and TGF-β intracellular pathway modulation by TRC105 in association with other tyrosine kinase inhibitors that did not show a functional synergistic effect (Figure 6C and 6D). We found that Sorafenib and Axitinib did not induce modulation of Akt and of the downstream Creb pathways, at variance of Sunitinib. Erk pathway was slightly modulated by Cabozantinib but the effect was reverted when in combination with TRC105, possibly due to Smad 2/3 activation of Erk (Figure 6C and 6D). Therefore, the specific effect of Sunitinib on Akt pathway appears involved in the synergy with TRC105-dependent phosphorylation of Smad 2/3, resulting in a significant reduction of Creb levels and of endothelial cell activation (Figures 6A, 6B and 7), hence, promoting endothelial cell death.

## DISCUSSION

Angiogenesis is a fundamental process required for the growth, development and expansion of solid tumors. In this work, we generated from renal cell carcinoma CSC-derived and tumor-derived endothelial cells expressing the surface glycoprotein CD105 (Endoglin) and tested the effect of TRC105, a CD105 monoclonal antibody, on the angiogenic properties of these tumor endothelial cells (TEC). We found that TRC105 alone inhibited the ability of TEC and CSC-TEC to organize in tubular structures, whereas it did not impair their proliferation or survival. Moreover, TRC105 increased the effect of the tyrosine kinase inhibitor Sunitinib in inhibiting tumor endothelial proliferation, survival and new vessel formation. The analysis of the molecular mechanisms involved in the combined effect of the two drugs showed simultaneous inhibition of TGF-β and VEGF signaling pathways with activation of Smad 2/3 apoptotic pathway and inhibition of Akt-dependent Creb activity, suggesting a synergism in promoting endothelial cell death.

TEC are the main targets of anti-angiogenic therapy [1] and may derive from recruited endothelial cells or from intra-tumor vasculogenesis due to endothelial differentiation of CSC [8]. However, the clinical activity of anti-angiogenic drugs has not been as robust as predicted by preclinical models. This may reflect angiogenic features of TEC compared to normal endothelium, and to the development of drug resistance from the activation of alternative intracellular pathways [32]. Indeed, endothelial cells treated with VEGF inhibitors overexpress angiogenic receptors possibly including CD105 [20]. Heterozygous CD105 expression and conditional knock-out of CD105 sensitize tumors to VEGF inhibition, suggesting that CD105 may be a mechanism of VEGF resistance [33]. TRC105 is a new anti-angiogenic monoclonal antibody targeting CD105. Indeed, CD105 is highly expressed on proliferating endothelial cells [23] and is of relevance in renal cell carcinoma, being expressed by CSC and CSC-derived TEC. In the present study, we confirmed our previous results [8, 31] showing that renal cell carcinoma-derived CD105^+^ cells possess stem cell properties, such as clonogenicity, sphere formation ability, *in vivo* tumorigenic ability and endothelial cell differentiation capability. We therefore investigated the effect of the CD105 targeting drug TRC105 on the angiogenic properties of renal TEC. Previous studies showed that CD105 inhibition affects tube formation but not the proliferation of normal endothelial cells (HUVEC) [34]. Our results confirmed that TRC105 alone also significantly inhibited tube formation but not viability of TEC. Moreover, TRC105 potentiated the effect of the anti-VEGF drug Sunitinib to inhibit proliferation, survival and tube formation of these cells. The combination of tyrosine kinase inhibition and TRC105 demonstrated a similar anti-angiogenic effect on normal endothelial cells, as reported [35]. This was notable given TEC are less sensitive to chemotherapy than normal endothelial cells [32]. Surprisingly, a combinatory effect was not observed on TEC with the anti-angiogenic drugs Axitinib, Sorafenib and Cabozantinib. This may suggest a specific interaction of the intracellular pathways blocked by Sunitinib and TRC105. The combined effect of tyrosine kinase and TGF-β inhibition was previously confirmed by *in vivo* experiments where the combination was effective in decreasing tumor vascular density, tumor growth and metastasis [33, 35]. TGF-β may induce both proliferation and apoptosis of endothelium, and CD105 acts as TGF-β co-receptor that influences the downstream pathways (Figure 7). In particular, activation of TGF-β receptor complex in the presence of CD105 results in phosphorylation of Smad 1/5, which activates cell growth [23]. In the absence of CD105, TGF-β signaling leads to Smad 2/3 phosphorylation and endothelial cell apoptosis [23]. In the present study, we found, accordingly, that CD105 inhibition by TRC105 on CSC-TEC promoted the phosphorylation of Smad 2/3 which, once in the nucleus, induces endothelial cell death. In addition, this effect was significantly increased when Sunitinib was used in combination with TRC105. The efficacy of this combination appears different from that observed in normal endothelial cells, where TRC105 blocked the TGF-β receptor-dependent phosphorylation of Smad 1/5 but it had no influence on Smad 2/3 [34]. Interestingly, TRC105 also enhanced the effect of Sunitinib on VEGF signaling, suggesting a molecular cooperation between the two drugs (Figure 7). It was recently reported that the combination of TRC105 and the tyrosine kinase inhibitor SU5416 might impair the direct interaction between CD105 and KDR [36]. Indeed, we show that the combination of TRC105 and Sunitinib decreased KDR levels and affected the phosphorylation of the VEGF-dependent transcription factor Creb in parallel to reduction of Akt phosphorylation. This may be reinforced by the additional effect of TRC105-mediated phosphorylation of Smad 2/3 on a direct Akt inhibition, as described [37]. Of note, the molecular pathways modulated by TRC105 and tyrosine kinase inhibition appear specific for the drug in use. In fact, in parallel with the absence of a functional synergistic effect, other tyrosine kinase inhibitors did not induce reduction of Akt-Creb pathway. Indeed, Cabozantinib, Axitinib and Sorafenib preferentially led to a slight modulation of the VEGF-R dependent Erk signaling pathway in TEC. The absence of synergy could be possibly explained by the described positive interaction of Erk on the TGF-β pathway, as reduced Erk activity could decrease Smad 2/3 phosphorylation and viceversa [38, 39]. No modulatory effect on Erk phosphorylation was instead observed in the presence of Sunitinib, as described in prostate tumor endothelial cells [40]. In conclusion, our results highlight a synergistic effect of TRC105 in combination with Sunitinib on tumor vascularization, proliferation and survival of CSC-TEC and TEC. This effect was greater than that observed on non-tumor endothelium and could be possibly due to high CD105 expression or activity. At a molecular level, we demonstrated that TRC105 and Sunitinib cooperate to promote cell apoptosis by inducing Smad 2/3 phosphorylation, through the TRC105-mediated inhibition of CD105, and by reducing the tyrosine kinase activity of KDR by Sunitinib. Both the decreased phosphorylation of Creb and the increased activity of Smad 2/3 may limit endothelial angiogenesis and induce apoptosis of endothelial cells (Figure 7). Interaction of VEGF and CD105 signaling was recently reported *in vitro* in endothelial cells [36] and *in vivo* in mouse models where CD105 targeting inhibited VEGF-R signaling [36] and altered the response of VEGF-R to VEGF [41]. Our results support this finding and indicate that the combined inhibition of these two pathways may have a potential use in renal cell carcinoma therapy.

## MATERIALS AND METHODS

### Cells isolation and characterization

Renal cell carcinoma stem cells (CSC) and primary carcinoma cell lines were isolated and characterized as previously described [8, 30, 31]. Briefly, CSC were obtained from specimens of renal cell carcinomas from patients undergoing radical nephrectomy according to the Ethics Committee of the S. Giovanni Battista Hospital of Torino, Italy (n. 168/2014). Magnetically sorted CD105^+^ CSC were cultured in the presence of the expansion medium, consisting of DMEM LG (Invitrogen), with insulin-transferrin-selenium, 10^-9^ M dexamethasone, 100 U penicillin, 1000 U streptomycin, 10 ng/ml EGF (all from Sigma-Aldrich) and 5% fetal calf serum (FCS) (Sigma-Aldrich) [8]. A CD105^+^ clonal renal cell carcinoma stem cell line was selected and used for all the experiments. Renal carcinoma primary cell lines were grown in DMEM and 10% FSC and CD105 expression was ranged from 2 to 20% (mean 11.49 %). TEC and HUVEC were obtained from renal cell carcinoma and from the umbilical vein respectively [32]. All endothelial cells were maintained in culture in EndoGRO MV-VEGF medium containing 10% FCS.

### Sphere formation

To grow CSC in non-adhesive condition as floating spheres, cells were plated at 1×10^5^ cells/mL in serum-free DMEM-F12 (Cambrex BioScience), supplemented with 10 ng/mL bFGF, 20 ng/mL EGF, 5 μg/mL insulin, and 0.4% bovine serum albumin (all from Sigma-Aldrich), as described [8, 31].

### Cytofluorimetric analysis

For cytofluorimetric analysis, cells were detached using a non enzymatic cell dissociation solution (Sigma-Aldrich) and resuspended in PBS 0.1% BSA (Sigma-Aldrich) and incubated with antibodies. The following antibodies, conjugated with fluorescein isothiocyanate (FITC), phycoerythrin (PE) or allophycocyanin (APC), were used: CD105, CD133, EPCAM, CD31, TIE-2, CD144 (Miltenyi Biotech) and SSEA4 (R&D).

### Endothelial differentiation of CSC

For endothelial differentiation, CSC were plated at a density of 50.000 cells/well into 6-well culture plated coated with Endothelial Cell Attachment Factor (Sigma-Aldrich), in EndoGRO (Merck Millipore) and maintained in hypoxia (1% O_2_ and 5% CO_2_) in hypoxia chambers (Stem Cells Technologies) for 14 days, as previously described [31]. CSC-derived TEC were maintained in culture for further 20 passages without observing any loss of phenotype.

### RNA isolation and Real time PCR

Total RNA was isolated from different cell preparations using Trizol Reagent (Ambion) according to the manufacturer's protocol. RNA was then quantified spectrophotometrically (Nanodrop ND-1000). For gene expression analysis, quantitative real-time PCR was performed. Briefly, first-strand cDNA was produced from 200 ng of total RNA using the High Capacity cDNA Reverse Transcription Kit (Applied Biosystems). Real-time PCR experiments were performed in 20-μl reaction mixture containing 5 ng of cDNA template, the sequence-specific oligonucleotide primers (purchased from MWG-Biotech) and the Power SYBR Green PCR Master Mix (Applied Biosystems). GAPDH or TATA-binding protein (TBP) mRNA were used to normalize RNA inputs. Fold change expression respect to control was calculated for all samples. The sequence-specific oligonucleotide primers used are GAPDH: forward, 5’-TGGAAGGACTCATGACCACAG T-3’ and reverse, 5’-CATCACGCCACAGTTTCCC-3’; E-CAD: forward 5’-GCATTGCCACATACACTCTCTTCT-3’, reverse 5’-GCTTGTTGTCATTCTGATCGGTTA-3’; MSI1: forward 5’-TTGGGAAGGTGGACGACG-3’, reverse 5’-CTCCACGATGTCCTCACTCTCA; VIM: forward, 5’-GGAACAGCA TGTCCAAAT CGA T-3’, reverse 5’-CAGCAAACTTGGATTTGTACCATT-3’; OCT4-A: forward 5’-AGCAGGAGTCGGGGTGG-3’, reverse, 5’-CTGGGACTCCTCCGGGTT-3’; KDR: forward 5’-GAACATTTGGGAAATCTCTTGCA-3’, reverse 5’-AGTCCAGAATCCTCTTCCATGCT-3’; PECAM: forward 5’-TGACAGTCAGAGTCATTCTTGCC-3’, reverse 5’-GGCTTTCCTCAGAAAATAACATTTG-3’; TIE2: forward 5’-CCCCTATGGGTGTTC-3’, reverse GCTTACAATCTGGCC-3’; VEGF: forward 5’-ATGAACTTTCTGCTCTCTTGGGTGC-3’, reverse 5’-TGATTCTGCCCTCCTCCTTCTGC-3’; ENG: forward 5’-TCACCACAGCGGAAAAAGG-3’, reverse 5’-GACACTCTGACCTGCACAAAGC-3’.

### CSC tumorigenic ability

In order to evaluate the vasculogenic potential of CSC, cells were implanted subcutaneously into SCID mice (Charles River) within growth factor–reduced Matrigel (BD Biosciences). Briefly, 1×10^3^ CD105+ CSC were re-suspended in 75 μl DMEM plus 125 μl of Matrigel at 4°C. Cells were injected subcutaneously into the left back of SCID mice (n=6). After 40 days, mice were sacrificed, and tumors recovered and processed for histology.

### Immunofluorescence

Immunofluorescence was performed on cryostatic sections of recovered CSC tumors for HLA class I polyclonal Ab and anti-human vWF (all from BioLegend). Sections were permeabilized with PBS-0.2% Triton for 6 minutes at 4°C. Alexa Fluor Texas Red goat anti-rabbit IgG and Alexa Fluor 488 goat anti-mouse IgG (all from Molecular Probes) were used as secondary antibody. Hoechst 33258 dye (Sigma-Aldrich) was added for nuclear staining. Confocal microscopy analysis was performed using a Zeiss LSM 5 Pascal model confocal microscope (Carl Zeiss).

### Anti-angiogenic drugs and reagents

Sunitinib malate, Cabozantinib, Sorafenib and Axitinib (Sigma-Aldrich) were resuspended in DMSO to a final concentration of 10 mM and stocked according to the manufacturer's instructions. Bevacizumab was purchased from Genentech and stored at +4°C. TRC105 was supplied by Tracon Pharmaceuticals, San Diego, CA and stored at +4°C.

### Proliferation

For proliferation assay, cells were plated in growth medium at a concentration of 2000 TEC-cells/well, 4000 CSC-derived TEC-cells/well in a 96-multiwell plate and left adhere overnight. The day after the culture medium was removed and a new medium containing different concentrations of drugs was added to the cells. DNA synthesis was detected after 48h as incorporation of 5-bromo-2-deoxyuridine (BrdU) using an enzyme-linked assay kit (Chemicon). Data are expressed as the mean ± SD of the media of absorbance of at least three different experiments, normalized to control (not treated cells).

### Apoptosis

Annexin V assay was performed using the Muse™ Annexin V & Dead Cell Kit (Millipore), according to the manufacturer's recommendations. Briefly, 25×10^3^ cells were incubated with different concentrations of drugs for 72 hours. Cells were then detached and resuspended in Muse™ Annexin V & Dead Cell Kit, and the percentage of apoptotic cells (Annexin V +) was detected.

### Tubulogenesis

*In vitro* formation of capillary-like structures was done on growth factor–reduced Matrigel (BD Biosciences). Cells (25×10^3^ cells per well) were seeded onto Matrigel- coated wells in EndoGRO MV-VEGF medium containing 10% FCS with or without TRC105, the indicated anti-angiogenic drug or the combination of both. Cells were periodically observed with a Nikon inverted microscope and experimental results recorded after 24 hours. Image analysis was performed with the ImageJ. Data were expressed as the mean ± SD of tube length in arbitrary units per field, normalized to untreated cells

### Drug targets array

Gene expression profiling was performed on tubule-like structures formed by 150×10^3^ CSC-TEC treated with vehicle (CTL), TRC105 (160 μg/ml), Sunitinib (0.1 μg/ml), or TRC105 and Sunitinib, using the RT^2^ Profiler™ PCR Array Human Cancer Drug Targets (PAHS 507Z, SA Biosciences). 200 ng of cDNA (synthesized using the RT2 First Strand kit (SABiosciences) according to manufacturer's instructions) were loaded for each CSC-TEC sample. The expression profile of 84 key drug targets genes was analyzed. Changes in the gene expression of treated, CSC-TEC were reported as a fold increase/decrease ±SD respect to untreated cells. Transcripts with a fold increase or decrease ≥2 were used for further investigation using Funrich V3 Software.

### Protein extraction and western blot

For protein analysis, cells were incubated with vehicle (CTL), TRC105 (160 μg/ml), Sunitinib (0.1 μg/ml), or TRC105 and Sunitinib for 3h and lysed at 4°C for 30 min in RIPA buffer (20 nM Tris·HCl, 150 nM NaCl, 1% deoxycholate, 0.1% SDS, 1% Triton X-100, pH 7.8) supplemented with protease and phosphatase inhibitors cocktail and PMSF (Sigma-Aldrich). Aliquots of the cell lysates containing 30 μg protein, as determined by the Bradford method, were run on 4-20%(Biorad) SDS-PAGE under reducing conditions and blotted onto PVDF membrane filters using the iBLOT system (Life Technologies). For Western blot analysis, anti-pCreb, anti-Creb, anti-pErk, anti-Erk, anti-pSmad 2/3, anti-pAkt, anti-Akt and anti-Vinculin Abs (all from Cell Signaling Technology) were used. Data are expressed as mean ± SD of the band intensity normalized with vinculin of three independent experiments.

### Statistical analysis

Statistical analysis was performed by using the Student *t* test, or ANOVA with Dunnet's multi-comparison tests, as appropriate. A p value of p<0.05 was considered significant.

## SUPPLEMENTARY MATERIALS FIGURES AND TABLE





## Abbreviations

ENG: Endoglin
TEC: tumor endothelial cells (deriving from total renal carcinomas)
CSC: cancer stem cells
CSC-TEC: tumor endothelial cells deriving from cancer stem cells
VEGF: vascular endothelial growth factor
VEGF-R: vascular endothelial growth factor-receptor
KDR: kinase insert domain receptor (VEGF-R2)
