# Supplementary material for: Effect of the monoclonal antibody TRC105 in combination with Sunitinib on renal tumor derived endothelial cells
Source: Oncotarget. 2018 Apr 27;9(32):22680–92. doi: 10.18632/oncotarget.25206 (PMC5978257; doi:10.18632/oncotarget.25206)
Supplement: Supplementary file 1 [file oncotarget-09-22680-s001.pdf]

Effect of the monoclonal antibody TRC105 in combination with Sunitinib on renal tumor derived endothelial cells

SUPPLEMENTARY MATERIALS

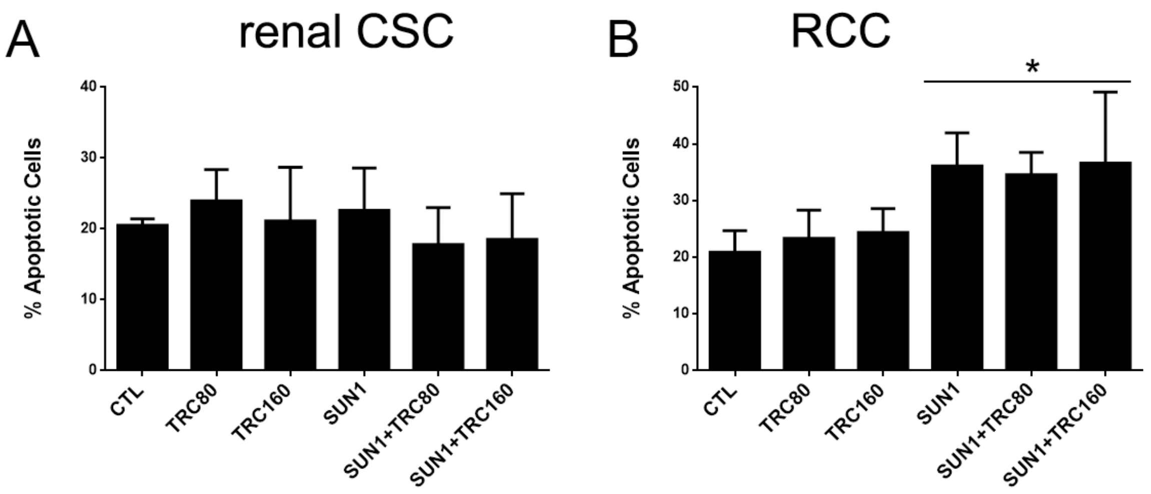

Supplementary Figure 1: Apoptosis assay of undifferentiated CSC and primary renal cell carcinoma cells (RCC) treated with TRC105 in combination with Sunitinib. Percentage of apoptotic CSC **(A)** or RCC **(B)** treated with Sunitinib alone (1µM) or in combination with TRC105 (TRC80-TRC160, corresponding to TRC105 80 and 160 µg/ml. *=$p<0.05$ vs CTL. Data are represented as mean ± SD of at least three independent experiments normalized to CTL.

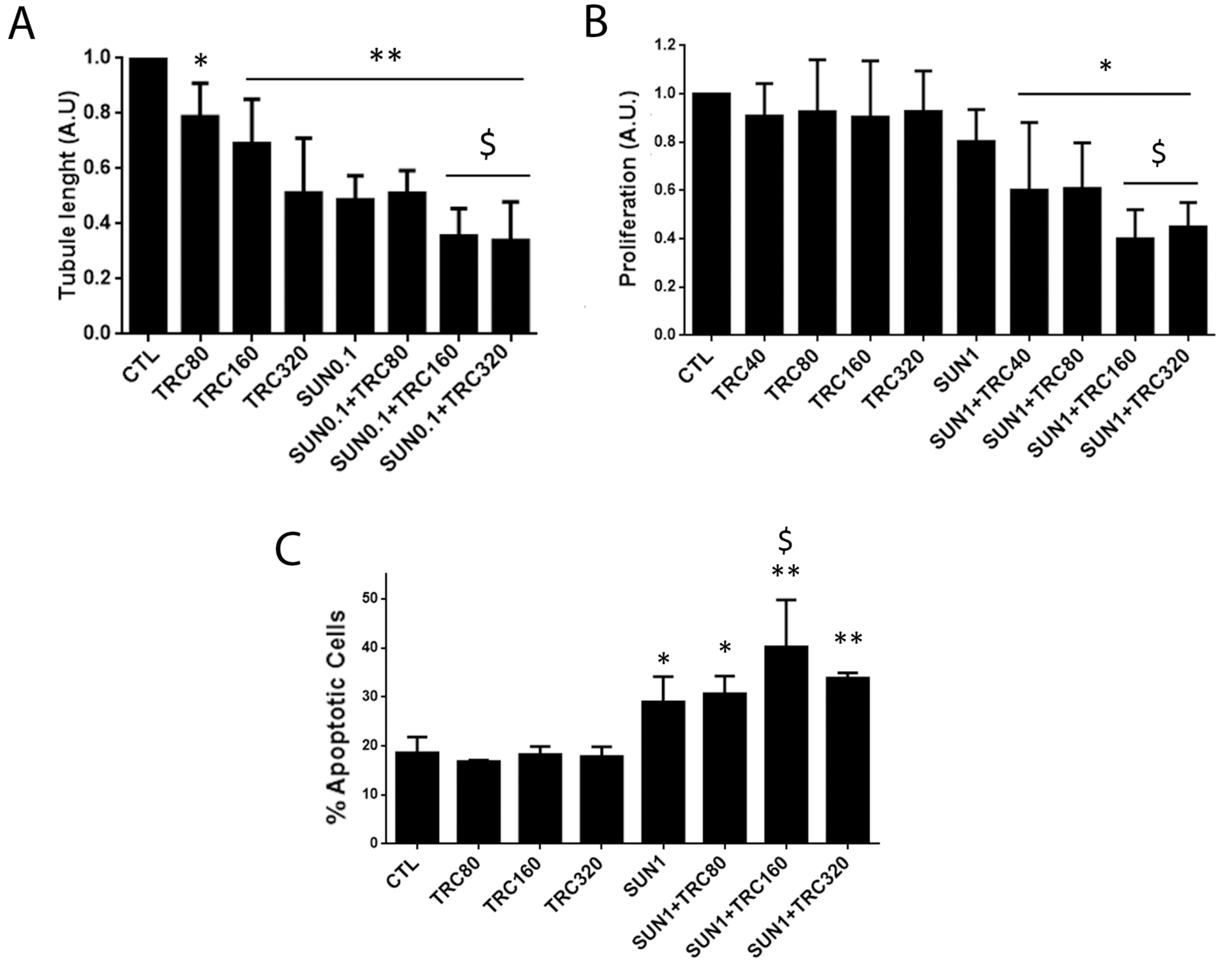

Supplementary Figure 2: Tubule formation, proliferation and apoptosis assay of HUVEC treated with escalating doses of TRC105 in combination with different anti-angiogenic drugs. (A) Tubule length of HUVEC treated with TRC105 (TRC80-320, corresponding to 80, 160 and 320 µg/ml) in combination with Sunitinib (SUN, 0.1 µM). *=p<0.05 vs CTL. **=p<0.001 vs CTL. $^{\$}$=p<0.001 vs TRC. **(B)** Proliferation of HUVEC treated with TRC105 (40, 80, 160 and 320 µg/ml) in combination with Sunitinib (SUN, 1µM). Data are represented as mean ± SD of at least three independent experiments normalized to CTL. *=p<0.05 vs CTL. $^{\$}$=p<0.001 vs TRC. **(C)** Percentage of apoptotic HUVEC treated with Sunitinib alone (SUN, 1µM) or in combination with TRC105 (80, 160 and 320 µg/ml). *=p<0.05 vs CTL. **=p<0.001 vs CTL. $^{\$}$=p<0.001 vs TRC. Data are represented as mean ± SD of at least three independent experiments normalized to untreated cells (CTL).

Supplementary Table 1: Fold increase/decrease expression of drug targets in CSC-TEC treated with TRC105 (160 µg/ml), Sunitinib (SUN, 0.1µM) or their combination (TRC+SUN), compared to untreated cells. Fold change <2 or >2 is reported. Sixteen genes regulated only by TRC+SUN are reported in red. Data are normalized to GAPDH and to untreated cells.

See Supplementary File 1
