# Supplementary material for: Effect of the monoclonal antibody TRC105 in combination with Sunitinib on renal tumor derived endothelial cells
Source: Oncotarget. 2018 Apr 27;9(32):22680–92. doi: 10.18632/oncotarget.25206 (PMC5978257; doi:10.18632/oncotarget.25206)
Supplement: Supplementary file 2 [file oncotarget-09-22680-s002.doc]

**Supplementary Table 1**

| **TRC105** | | **SUN** | | **TRC+SUN** | |
| --- | --- | --- | --- | --- | --- |
| **GENE** | **FOLD CHANGE** | **GENE** | **FOLD CHANGE** | **GENE** | **FOLD CHANGE** |
| TXN | 5.86 | TXN | 2.44 | ERBB3 | 6.88 |
| HSP90B1 | 5.22 | ESR2 | 2.26 | AURKC | 6.23 |
| HDAC2 | 5.1 | IRF5 | -2.74 | TXN | 4.4 |
| PIK3C3 | 4.95 | HDAC11 | -2.93 | PIK3C3 | 3.61 |
| RHOA | 4.95 | PRKCB | -3.22 | PGR | 3.02 |
| CDK7 | 4.91 |  |  | PDGFRA | 2.94 |
| PARP4 | 4.9 |  |  | PARP4 | 2.89 |
| CTSS | 4.88 |  |  | PRKCA | 2.85 |
| CDK1 | 4.43 |  |  | CTSS | 2.82 |
| HSP90AA1 | 4.34 |  |  | HDAC2 | 2.76 |
| PARP2 | 4.09 |  |  | HSP90B1 | 2.71 |
| TP53 | 3.92 |  |  | CDK7 | 2.70 |
| PIK3CA | 3.7 |  |  | PRKCE | 2.69 |
| PRKCA | 3.64 |  |  | CDK1 | 2.57 |
| BIRC5 | 3.6 |  |  | ATF2 | 2.52 |
| PLK4 | 3.49 |  |  | EGFR | 2.48 |
| CDK8 | 3.37 |  |  | TOP2B | 2.44 |
| ATF2 | 3.28 |  |  | PARP2 | 2.41 |
| CTSL | 3.18 |  |  | PLK2 | 2.36 |
| EGFR | 3.11 |  |  | HDAC6 | 2.34 |
| NRAS | 3.09 |  |  | RHOA | 2.34 |
| PRKCE | 2.99 |  |  | HDAC1 | 2.31 |
| HDAC8 | 2.94 |  |  | ERBB4 | 2.29 |
| PIK3C2A | 2.94 |  |  | TERT | 2.29 |
| TOP2A | 2.92 |  |  | AKT2 | 2.28 |
| CTSB | 2.81 |  |  | TOP2A | 2.27 |
| CTSD | 2.75 |  |  | KRAS | 2.24 |
| AKT2 | 2.55 |  |  | CTSL | 2.23 |
| KRAS | 2.53 |  |  | FIGF | 2.19 |
| MTOR | 2.52 |  |  | PLK4 | 2.17 |
| GSTP1 | 2.47 |  |  | PIK3CA | 2.16 |
| PLK2 | 2.42 |  |  | CDK9 | 2.11 |
| PTGS2 | 2.42 |  |  | BIRC5 | 2.08 |
| CDK2 | 2.37 |  |  | CDK8 | 2.07 |
| HDAC1 | 2.36 |  |  | GRB2 | 2.06 |
| AURKA | 2.29 |  |  | CTSB | 2.02 |
| HDAC6 | 2.22 |  |  | MDM2 | -2.03 |
| TNKS | 2.19 |  |  | PRKCB | -2.08 |
| HDAC4 | 2.17 |  |  | KDR | -2.11 |
| HRAS | 2.14 |  |  | IGF2 | -2.16 |
| TOP2B | 2.03 |  |  | FLT1 | -2.29 |
| HDAC11 | -2.35 |  |  | FLT4 | -2.29 |
| PRKCB | -2.59 |  |  | IGF1 | -2.29 |
| MDM2 | -2.97 |  |  | KIT | -2.29 |
| ERBB2 | -3.82 |  |  | MDM4 | -2.32 |
| HIF1A | -3.92 |  |  | HIF1A | -2.49 |
|  |  |  |  | HDAC11 | -5.31 |

**Supplementary Table 1:** Fold increase/decrease expression of drug targets in CSC-TEC treated with TRC105 (160 μg/ml), Sunitinib (SUN, 0.1μM) or their combination (TRC+SUN), compared to untreated cells. Fold change <2 or >2 is reported. Sixteen genes regulated only by TRC+SUN are reported in red. Data are normalized to GAPDH and to untreated cells.
